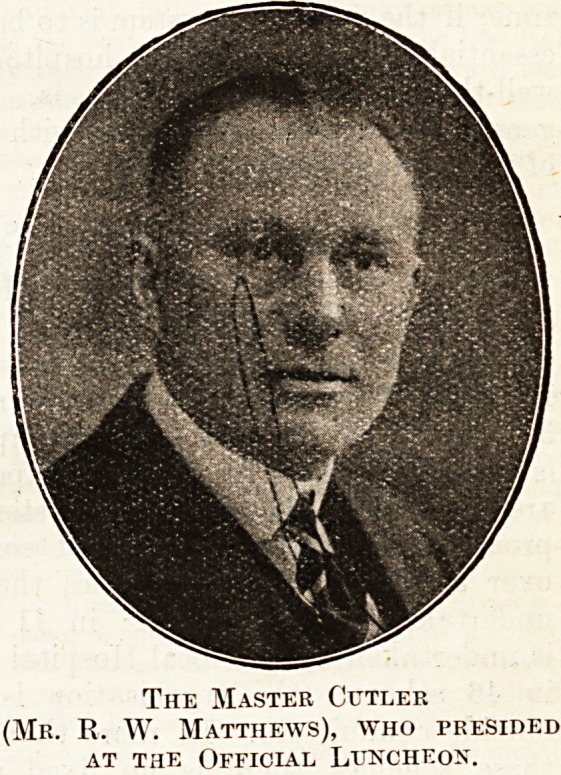# Sheffield Hospital Conference

**Published:** 1923-07

**Authors:** 


### SHEFFIELD HOSPITAL CONFERENCE.

### AN IMPRESSION BY A RANK=AND=FILE MEMBER.

ALL the London hospital world and (in some instances) his wife was leaving the Midland and Great Central Railway stations on Wednesday night, May 30, and no doubt a similar exodus towards Sheffield might have been observed at each of the great centres. Certainly the 1923 Congress of the British Hospitals Association was, in point of numbers if not in all other respects, a record. When, with business-like punctuality, Sir Arthur Stanley opened the proceedings at 10 o'clock on the Thursday morning, the large and badly ventilated Conference room at the Royal Victoria Hotel was full to an almost uncomfortable extent.

Sir Henry Hadow, C.B.E., Vice-Chancellor of Sheffield University, who presided at the Official Dinner.

A civic welcome was given by Alderman Charles Simpson, the Deputy Mayor, who in the course of his remarks referred to the great success of the penny-a-week scheme organised by the Joint Hospitals Council, which more than once has received detailed notice in The Hospital and Health Review. The revenue from this scheme has now reached approximately £100,000 a year. On the principle of business before pleasure, the meeting then discussed a suggestion for a Central Fund for the Provinces (on the lines of King Edward's Fund), and the delegates rejected it as decidedly as they had accepted the principle, and asked for further enlightenment, at the Liverpool Congress of 1922. The provincial members were obviously a little uneasy about the possible interference of London.

Dr. H. L. Eason's paper on "Problems of Hospital Administration" was the next item on the programme. It was an excellent paper, bristling with useful hints, shrewd remarks, witty sallies, and unusual literary allusions. Let us hope that it may find a larger public. Here are some of Dr. Eason's *obiter dicta*:—"In reference to contributory schemes, those connected must understand that they will never get out of the hospital as much as they can put in." "Medical men on the Board of Management are sometimes both servants and masters." "If your works department is not behind in its work it is probably too large." "Nurses' salaries have gone up and will never come down again." "A simple gas-ring in a ward kitchen may cost more than three coal fires." "The modern captains of industry—the descendants of the robber-barons of the Middle Ages."

The Earl of Onslow, Chairman of the Voluntary Hospitals Commission.

Dr. S. S. Goldwater, of Mount Sinai Hospital, New York, joined in the discussion. He referred to a question put to the American Hospitals Association at one of its meetings: "Who is the most important person in a hospital?" Various answers were given, and doubtless some solutions remained unspoken. The answer was: "The patient." In the afternoon Sir Napier Burnett read a paper bristling with facts and figures on "Voluntary Hospital Finance," and Viscount Hambleden followed with the latest aspects of his favourite subject, "Hospital Finance in its Relation to Approved Societies." He was unable to explain why great societies like the Hearts of Oak and the Oddfellows were unable to join with the Prudential and others in meeting obligations to hospitals. One thing became evident during the Conference, and that was that at future meetings of the British Hospitals Association it would be necessary to put a time limit to speeches. Indeed, one or two speakers (not readers of papers) long outstayed their welcome. One of them spoke at the rate of quite 200 words a minute, and must have provided excellent exercise for the reporters.

In the afternoon there was a reception at the Town Hall by the Lady Mayoress, and in the evening a dinner given by Sheffield to the visiting members of the British Hospitals Association. Sir

[*Russell.*

Viscount Hambleden.

Henry Hadow, Vice-Chancellor of Sheffield University, took the Chair. During the evening Sir Arthur Stanley said he did not tip his brother, Lord Derby's, horse for the Derby, but "the family were going to Epsom on Wednesday to see what happened." This light relief to the grave debates of the day was especially welcomed by the company. Mr. S. R. Lamb, Secretary of the Joint Hospitals Council, was indefatigable in ensuring the comfort of all the visitors, and the success of the gathering. With his staff he undoubtedly worked very hard indeed. Therefore Mr. Courtney Buchanan's remark at the dinner that he felt "like a wolf in sheep's clothing" brought the house down.

On the second morning the *pièce de résistance* was Mr. H. J. Waring's Paper on "The Future of Voluntary Hospitals from a Medical Viewpoint." Mr. Waring has his eye (like all far-seeing people) upon the American hospital for all classes as the first solution to our troubles. Dr. Arthur Hall, of the Royal Infirmary, Sheffield, in an excellent speech, outlining what he considered to be the fundamental principles in any changes to be made in the hospital system, said that the task would be easier if no voluntary hospitals existed at all. If they could start *de novo*, he was sure that some of them could conceive a number of excellent schemes—on paper. Voluntary hospitals, however, did exist, and they were somewhat in the position of a man living in a comfortable house who wanted additions and alterations in order to bring it into line with modern requirements; any attempt to do that was difficult and always expensive. But there was one thing they must do—that was to avoid the destruction of good features. Alluding to good features of voluntary hospitals of to-day, which he would not like to see destroyed, Professor Hall suggested that the first was the esprit de corps which was developed to quite a surprising degree. From the Chairman of the Board down to the youngest person they got extreme loyalty to the institution. That was an invaluable asset, not only to a hospital, but to a sick public, and consequently the nation. But it was the extreme loyalty which made it extraordinarily difficult to get the hospitals to co-operate; it was part of the barrier which made co-operation and re-organisation extremely difficult. The second good feature which he would like to see preserved was the medical efficiency which he regarded as synonymous with progress. All the progress that had been made during the past 100 years had come from the large voluntary hospitals. With regard to esprit de corps and efficiency, he declared that the teaching centres were the strokes to the hospital eights. The third feature which he did not want to see destroyed was what had been described by Mr. Waring as equality in the wards. As soon as a patient went into the ward of a voluntary hospital he got exactly the same proportional treatment according to the gravity of his disease. Speaking generally, throughout the country it was recognised as something which did exist. It was not the monopoly of the voluntary hospital. The same thing was found in the hospitals under other systems, but it began in the voluntary hospitals. Those three things together made up something which he would speak of as the spirit of the hospitals. It was something intangible, ethereal—a precious legacy from their forefathers. Whatever they did in the way of alteration it was most important that they should not destroy that. He was sanguine enough to think that they could bring new methods into the hospitals without permitting the hospital spirit to fly out of the window.

Alderman Wm. C. Fenton (Lord Mayor of Sheffield).

The Master Cutler (Mr. R. W. Matthews), who presided at the Official Luncheon.

The chief figures of the Congress were entertained at lunch by the Master Cutler (who wore his magnificent jewel) at Cutlers' Hall, and received excellent hospitality amid the associations and records of the ancient Guild. In the afternoon the visitors were received by the hospitals of the city, and later a number were taken motor drives through the beauty spots of the district. Some of the members were glad to accept an arrangement to stay at the Guest House, Froggatt Edge, near Grindleford. This gathering, with those from Sheffield specially interested, joined in an informal debate, arranged for Saturday evening, which was opened by Miss Cummins on the subject of "The Almoner System in Hospitals" —a summary of her paper will be found on page 281. This discussion proved of great interest to those who heard it. On Sunday practically the last members of the Conference dispersed to their own homes after four days of instruction, entertainment and unbroken good fellowship.

---

Dr. T. W. Naylor Barlow, of Wallasey, has been elected president of the Society of Medical Officers of Health.

## Figures and Tables

**Figure f1:**
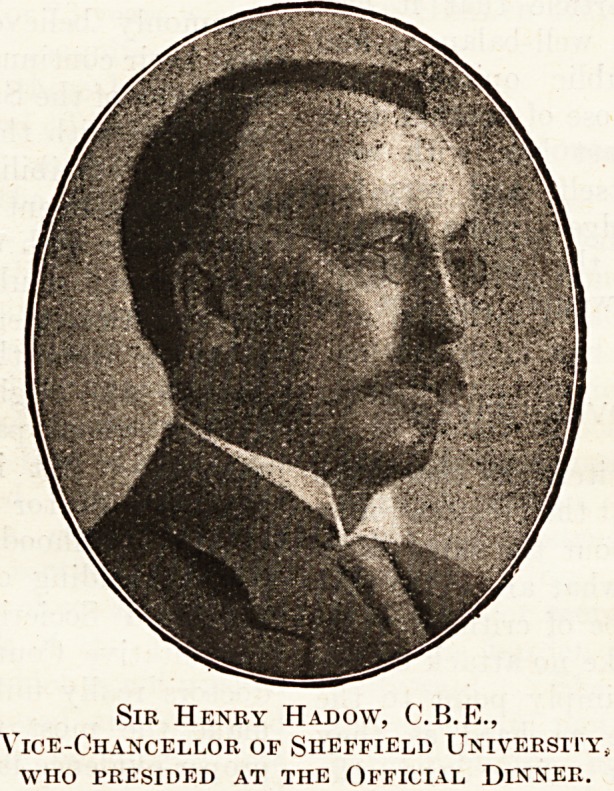


**Figure f2:**
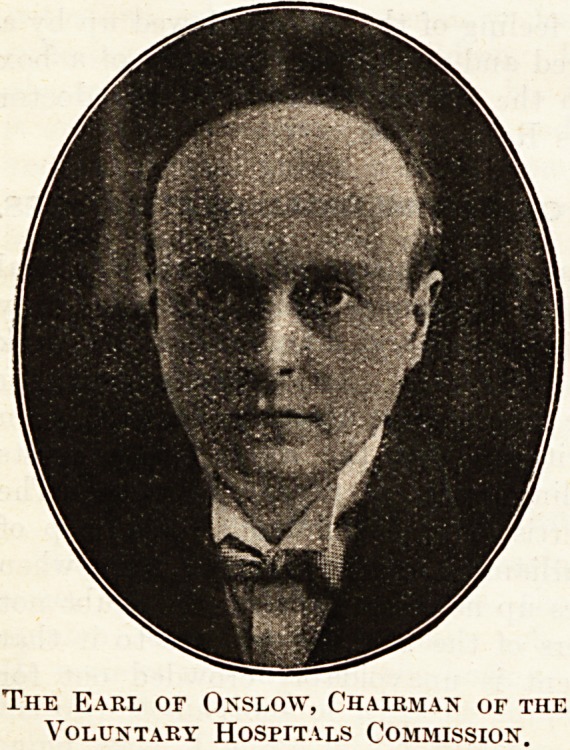


**Figure f3:**
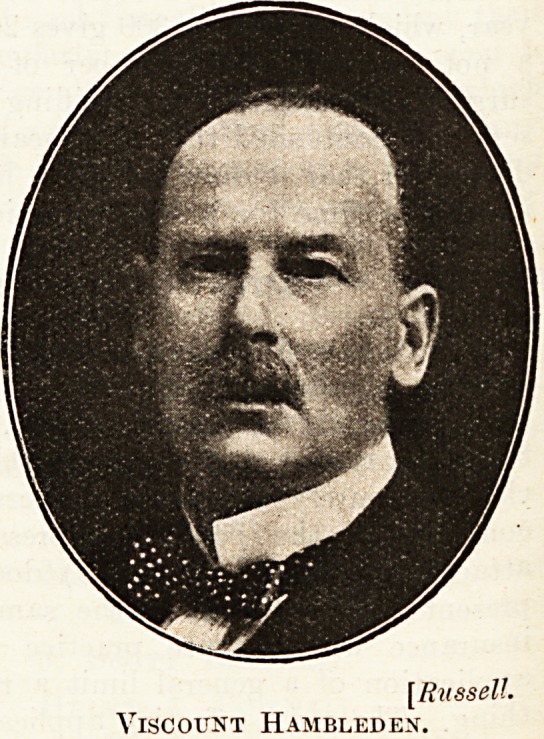


**Figure f4:**
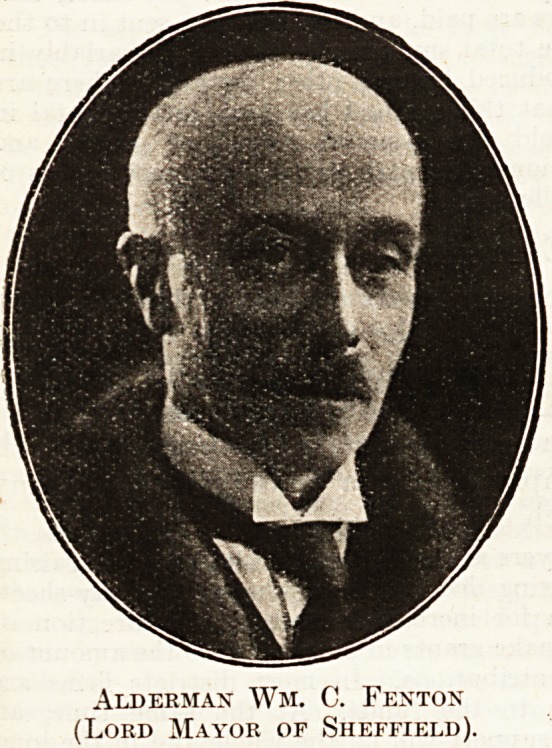


**Figure f5:**